# Supplementary material for: Identification of photoperiod-induced specific miRNAs in the adrenal glands of Sunite sheep (Ovis aries)
Source: Front Vet Sci. 2022 Jul 22;9:888207. doi: 10.3389/fvets.2022.888207 (PMC9354845; doi:10.3389/fvets.2022.888207)
Supplement: Supplementary file 1 [file Data_Sheet_1.ZIP › Supplementary file/miRDeep2 file.zip!/miRDeep2 file/Novel_Structure/SPLP42A1/Novel_316.pdf]

Provisional ID	:	NC_019464.2_12701
Score total	:	2.1
Score for star read(s)	:	-1.3
Score for read counts	:	0
Score for mfe	:	1.8
Score for randfold	:	1.6
Score for cons. seed	:	
Total read count	:	103
Mature read count	:	103
Loop read count	:	0
Star read count	:	0

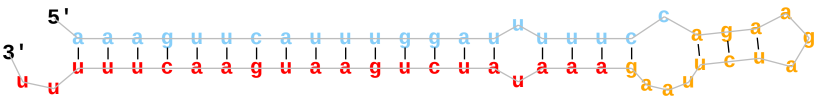

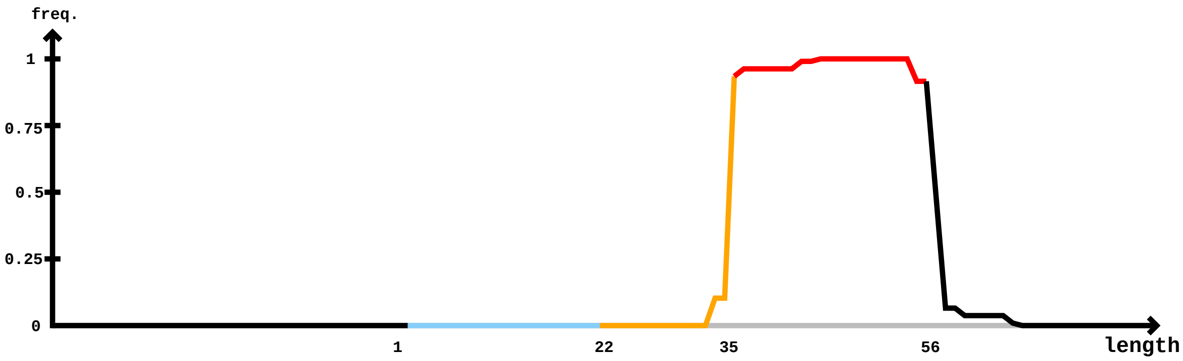

Star   Mature

```
5'- ugucauaaaaggguaaauacuguugaguuggucaaaaaguucauuuggauuuuuccagaagaucuuaagaaauaucugaaugaacuuuuuggccaacccgauuuauaauucca   -3'    exp
    ..........(((...(((..((((.((((((((((((((((((((((((.((((.(((...)))...)))).)))))))))))))))))))))))).)))).)))...))).    reads      mm         sample
    ................................agaaaAaucugaaugaacuuuuu..........................................................    11         1          seq
    ..................................aaaAaucugaaugaacuuu............................................................    9          1          seq
    ..................................aaaAaucugaaugaacuuuuu..........................................................    80         1          seq
    ...................................aauaucugaaugaacuuuuuggc.......................................................    1          0          seq
    ...................................aaAaucugaaugaacuuuuuggc.......................................................    2          1          seq
    .........................................ugaaugaacuuuuuggccaacc..................................................    1          0          seq
    .........................................ugaaugaacuuuuuggccaacA..................................................    1          1          seq
    .........................................ugaaugaacuuuuuggUcaaccc.................................................    1          1          seq
    ...........................................aaugaacuuuuuggccaacc..................................................    1          0          seq
```
